# Predictors of successful weaning from veno-arterial extracorporeal membrane oxygenation (V-A ECMO): A systematic review and meta-analysis

**DOI:** 10.1371/journal.pone.0310289

**Published:** 2025-03-19

**Authors:** Henry R. Hsu, Praba Sekhar, Jahnavi Grover, David H. Tian, Ciaran Downey, Ben Maudlin, Chathuri Dissanayake, Mark Dennis

**Affiliations:** 1 School of Medicine, University of Sydney, Sydney, New South Wales, Australia; 2 Department of Anaesthesia and Perioperative Medicine, Westmead Hospital, Sydney, New South Wales, Australia; 3 School of Medicine, Western Sydney University, Sydney, New South Wales, Australia; 4 Department of Anaesthesia, Royal Prince Alfred Hospital, Sydney, New South Wales, Australia; 5 Department of Anaesthesia, Royal North Shore Hospital, Sydney, New South Wales, Australia; 6 Department of Intensive Care, Royal Prince Alfred Hospital, Sydney, New South Wales, Australia; 7 Department of Cardiology, Royal Prince Alfred Hospital, Sydney, New South Wales, Australia; Azienda Ospedaliero Universitaria Careggi, ITALY

## Abstract

**Background:**

Venoarterial extracorporeal membrane oxygenation (V-A ECMO) use to support patients in cardiac failure is increasing. Despite this increased use, predicting successful weaning from ECMO can be challenging, no uniform guidelines on weaning exist. Therefore, we completed a systematic review to evaluate prognostic factors that predict successful weaning from V-A ECMO.

**Methods:**

Following the PRIMSA guidelines, a systematic literature search of Medline, Embase, SCOPUS and CENTRAL identified original research studies of patients requiring V-A ECMO where weaning was attempted. Data was collected on demographic factors and weaning protocol, biomarkers, haemodynamic, echocardiographic factors for the successfully weaned (SW) and not successfully weaned (NSW) groups. Two investigators reviewed studies for relevance, extracted data, and assessed risk of bias using the ROBINS-I tool. The study was registered on the international prospective register of systematic reviews (PROSPERO ID# CRD42022366153).

**Results:**

1219 records were screened, of which 20 studies were deemed sufficient to be included in the statistical analysis based on pre-specified criteria. Factors associated with successful weaning were higher left ventricular ejection fraction (LVEF) (MD 9.0, 95% CI 4.1–13.8; p < 0.001) and left ventricular outflow tract velocity time integral (LVOT VTI) at time of weaning, (MD 1.35, 95% CI 0.28–2.40 lactate at admission (MD −3.2, 95% CI −4.8 to −1.5, p < 0.001), and CK-MB at admission (MD −4.11, 95%CI −6.6 to −1.6, p = 0.001). Critical appraisal demonstrated moderate-high risk of bias owing to confounding and low sample sizes.

**Conclusion:**

In patients on V-A ECMO support being assessed for weaning multi-parametric assessment is required. Moderate-high heterogeneity and low sample sizes warrant higher-quality studies to help guide decisions to wean patients from V-A ECMO.

## Introduction

Veno-arterial (V-A) extracorporeal membrane oxygenation (ECMO) is increasingly being used [1,2] in acute cardiac failure to provide end-organ perfusion whilst definitive treatment, myocardial recover occurs or bridge to left ventricular assist device (LVAD) or heart transplant is completed.

Complications whilst on V-A ECMO support are common and effect mortality and increase with duration of support [3]. Therefore, minimising duration of V-A ECMO support, where possible, is sought. However, premature withdrawal of V-A ECMO support, may result in recurrence of cardiogenic shock and effect recovering organs. Minimizing complications associated with device support with the potential for hemodynamic deterioration if support is prematurely discontinued can be challenging.

The definition of successful V-A ECMO weaning has been proposed as when a patient survives for longer than 48 hours after ECMO explantation, with more, recent definitions as those having ECMO removed and not requiring further mechanical support because of recurring cardiogenic shock over the following 30 days [4,5]. Depending on the definition the proportion of V-A ECMO patients successfully weaned ranged between 30–75% [4–9].

A variety of clinical, haemodynamic, biochemical and echocardiographic variables have been proposed and used to guide clinical improvement and readiness to wean [10]. However, criteria and variables have not been completely reviewed to ascertain effectiveness [11] and meta-analyses as yet not completed. Therefore, we systematically reviewed a broad range of biomarkers, haemodynamic, echocardiographic and scoring systems to predict successful weaning from V-A ECMO.

## Materials and methods

The study was conducted as previously outlined in our registered and published protocol (PROSPERO ID# CRD42022366153) and in accordance with the PRISMA (Preferred Reporting Items for Systematic Reviews and Meta-Analyses) guidelines [12]. Ethics approval and patient consent were not required. The PRISMA checklist is provided in S1 Appendix.

### Search strategy

The search strategy is detailed in a Material. A comprehensive search of three electronic databases (Medline, Embase, SCOPUS and CENTRAL) was conducted in October 2022, which were re-run in December 2023 and March 2024 prior to final analysis and further studies retrieved for inclusion. Appropriate Boolean operators were used to combine search terms that included V-A ECMO, ECMO, extra-corporeal life support, weaning, decannulation and ECLS. The reference lists of all included studies were also reviewed to identify any additional articles, and duplicate articles were removed. Studies that were not primarily in the English language were included if they were accompanied by an English translation. There were no limitations on the publication period. The search strategy is provided in S2 Appendix.

### Study characteristics

Inclusion criteria allowed for randomized controlled trials, cohort studies, case series and conference abstracts that (1) considered adult or paediatric populations (2) involved patients who were on V-A ECMO and (3) there was an attempt to de-cannulate/wean from ECMO. Studies using ECMO as a bridge to ventricular assist device or heart transplant were excluded. Case series were included if >5 patients. Studies had to report associations between variables within the study and weaning success. Review publications, grey literature, non-English language publications, editorials, comments, letters to the editor and animal studies were excluded. Studies only assessing baseline variables with weaning success were excluded.

### Study selection

Title and abstract screening were conducted by independent investigators (P.S. or H.H. or C.D.). Likewise, full-text screening was performed by two independent investigators (P.S. or H.H. or C.D.). All conflicts were resolved by a third, senior investigator (M.D.). The systematic review platform Covidence (www.covidence.org; Veritas Health Innovation, Melbourne, Australia) was used to facilitate the screening process. Publications found to fulfil eligibility criteria underwent data extraction. Literature search and decisions are provided in S1 Table.

### Data extraction

Data was extracted from studies by two independent reviewers (P.S. or H.H. or C.D. or J.G.) using Microsoft Excel. Extracted variables included but not limited to patient demographics, weaning protocol, successful weaning definition, weaning success, various prognostic factors including biomarkers, haemodynamic, echocardiographic and scoring systems. The primary outcome was weaning success defined survival post removal of mechanical circulatory support and not requiring ventricular assist device or heart transplant. Meta-analysis was planned of predictors as appropriate. Missing data was reported as not reported. Authors were attempted to be contacted for further or missing data via email. Data extraction table is provided in S2 Table.

### Evaluation of risk of bias

Critical appraisal of the risk of bias for individual studies was conducted using the ROBINS-I Tool (Risk of Bias in Non-Randomized Studies - of Interventions) [13]. Each included study was scored by two independent investigators (P.S. or J.G. or H.H.). Any discrepancies between the two reviewers were resolved by discussion and mutual agreement. Studies of poor-quality following risk of bias assessment were not be excluded from being included in our synthesis. Where a poor-quality study has contributed to a synthesized effect estimate, we explored the impact of study quality by performing sensitivity analysis by removing the poor-quality study to observe the impact that bias has had on the synthesized effect. Risk of bias assessment is provided in S3 Table.

### Statistical analysis

Meta-analysis was completed as per the Cochrane Handbook for Systematic Reviews of Interventions when the outcomes were reported by two or more trials [14]. Statistical analysis was performed using Review Manager (version 5.3, The Cochrane Collaboration, Oxford, UK). For continuous outcomes, mean, standard deviation (SD) and sample size were extracted from each of the groups. Where studies reported median and ranges or interquartile range, derived mean and standard deviation as described by Wan et al. were calculated [15]. Mean differences with 95% confidence interval (CI) were used for continuous outcomes. An inverse variance method was applied for mean difference. Heterogeneity was assessed using I^2^ statistics and values between 50% and 90% were considered to represent substantial heterogeneity. A random effects meta-analysis model and exploring heterogeneity with sensitivity and subgroup analysis were applied where appropriate. Categorisation of reported risk factors of successful weaning from studies that reported multivariable adjustment was completed.

## Results

### Systematic search and study selection

**Fig 1:**
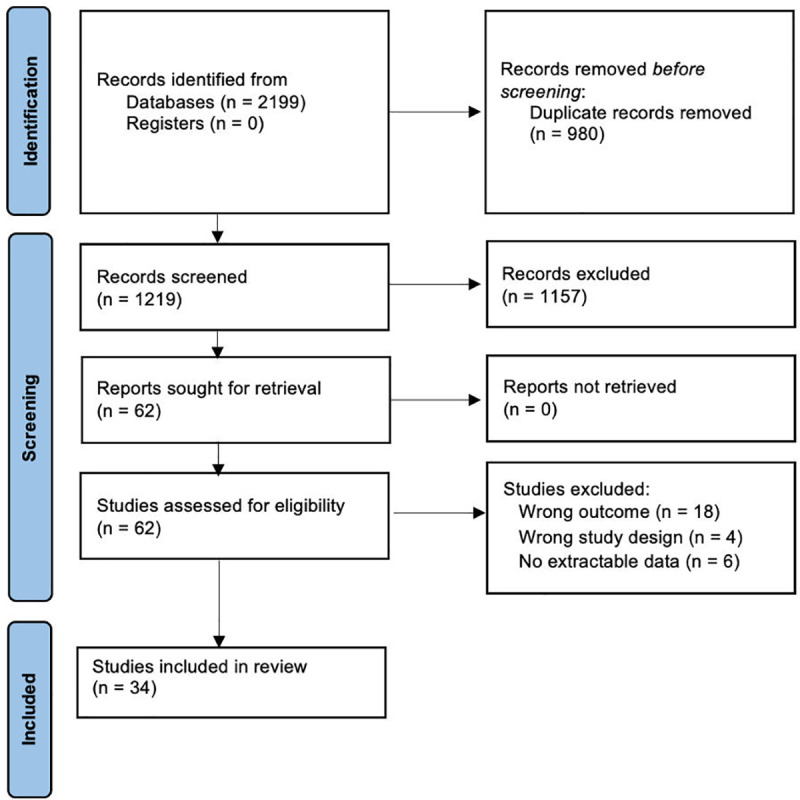
PRISMA flow diagram illustrating the number of studies identified by the search and the stages in which they were chosen and eliminated.

**Fig 2:**
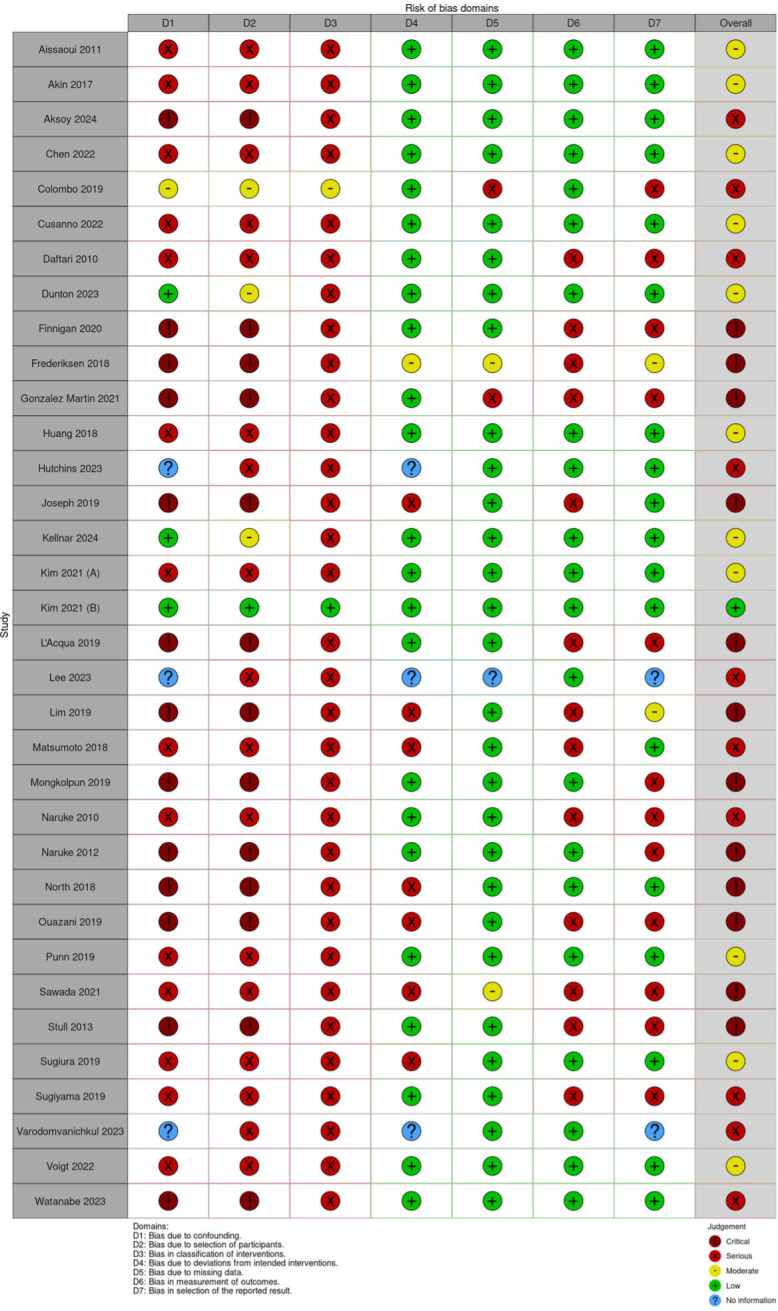
Risk of bias of the included studies (ROBINS-I).

The search strategy of relevant references yielded a total of 2199 references (Fig 1). After the removal of 980 duplicates, the remaining 1219 references were screened by title and abstract. A total of 62 publications were deemed to be eligible for full-text screening, of which 28 studies were excluded with reasons. A total of 34 articles were included in the final analysis, of which 20 studies were deemed sufficient to be included in the statistical analysis. Risk of bias assessment is summarised in Fig 2. The remaining 14 studies examined differing prognostic predictors that were not able to be meta-analysed together. The sample sizes ranged from 12 to 265 patients, with a pooled sample size of 1903 patients. Twenty-six publications were retrospective cohort studies, seven were prospective cohort studies, and one not recorded. Cardiogenic shock was the primary indication for V-A ECMO in ten publications, myocarditis in two, cardiomyopathy in two, cardiac arrest in four, pulmonary embolism in one, post cardiac surgery in two, congenital heart disease in one, acute respiratory distress syndrome in one, and eleven not recorded. Geographically, fourteen publications were from Asia, eight were from North America, eleven were from Europe, and one was from the Middle East. A summary of the baseline characteristics of included papers and clinical variables is provided in Table 1.

**Table 1:** Characteristics of included studies.

Author	Country	Enrolment period	Type of study	Total number of patients	Successful weaned	Unsuccessful wean	Age		Male		BMI		Heart failure (co-morbidity)	Indication for V-A-ECMO (%)	Successful weaning definition	Weaning success (%)
							Success wean	Unsuccess wean	Success wean	Unsuccess wean	Success wean	Unsuccess wean				
Aissaoui 2011 [16]	France	2007–2008	Prospective cohort	38	25	13	49 ± 14	67 ± 11	25	8	NR	NR	8	CMP (47%), FM (6%), Post-cardiotomy shock (22%), Post-transplantation (10%), Other (16%)	ECMO remoV-Al and no further MCS because of recurring CS over the following 30 days	20/38 = 53%
Akin 2017 [17,29]	Netherlands	2014–2016	Prospective cohort	13	10	3	56 ± 17	41 ± 16	9	1	NR	NR	0	PE (38%), Post-cardiotomy shock (23%), CS post-AMI (15%), Myocarditis (15%), Intoxication (8%)	Successful V-A-ECMO explantation within 48 h	10/13 = 77%
Aksoy 2024 [18]	Turkey	2010–2019	Retrospective cohort	55	27	28	2.3	1.6	19	20	NR	NR	NR	Post-op complications following congenital heart surgery, including: low cardiac output syndrome, inability to wean from bypass, ECPR.	Wean trial when adequate myocardial contraction and haemodynamically stable; initiated with flow rate to 25%	27/55 = 49%
Chen 2022 [19]	Taiwan	NR	Retrospective cohort	47	31	16	69 ± 16	39 ± 18	15	9	NR	NR	NR	NR	Weaning from ECMO and surviV-Al beyond 48 h	31/47 = 66%
Colombo 2019 [20]	Italy	2013–2017	Retrospective cohort	25	18	7	NR	NR	NR	NR	NR	NR	NR	CPR (71%), CS post-AMI (17%), Myocarditis (7%), PE (4%), Takotsubo (2%), Intoxication (2%)	Device remoV-Al without requirement for re-cannulation over the following 30 days	18/25 = 72%
Cusanno 2022 [9]	France	2016–2021	Retrospective cohort	57	36	21	46 ± 19	57 ± 17	NR	NR	26 ± 6	25 ± 7	37	Ischemic CS (35%), Refractory CA (28%), Other (37%)	NR	36/57 = 63%
Daftari 2010 [21]	USA	2000–2008	Retrospective cohort	27	16	11	NR	NR	NR	NR	NR	NR	27	Heart failure (100%)	NR	16/27 = 59%
Dunton 2023 [22]	USA	2016–2021	Retrospective cohort	265	140	125	59.1 ± 13.6	59.4 ± 13.2	95	100	30.8 ± 8.1	30.4 ± 7.4	75	CPR, cardiogenic shock, post cardiotomy shock	Survival to decannulation	140/265 = 53%
Finnigan 2020 [23]	United Kingdom	NR	NR	14	NR	NR	NR	NR	NR	NR	NR	NR	NR	Post cardiac surgery support (57%), Respiratory support (43%)	NR	NR
Frederiksen 2018 [24]	Denmark	NR	Retrospective cohort	29	15	14	NR	NR	NR	NR	NR	NR	NR	NR	ECMO weaning and being alive 24 h later without hemodynamic MCS	15/29 = 52%
Gonzalez Martin 2021 [25]	Spain	2013–2020	Retrospective cohort	85	52	33	NR	NR	NR	NR	NR	NR	0	CS (47%), ECPR (9%), Electrical storm (9%), Post-cardiotomy CS (33%), Other (1%)	Survival >24 h after explant and no mortality from cardiogenic shock/heart failure or cardiac arrest during admission	52/85 = 61%
Huang 2018 [26]	Taiwan	2014–2015	Retrospective cohort	46	28	18	59 ± 16	48 ± 15	18	15	NR	NR	8	CS/Cardiac arrest post AMI (50%), Dilated cardiomyopathy (15%), VT/VF Arrest (11%), Myocarditis (8%), PE (4%)	ECMO removal and no mortality and/or MCS because of recurring CD over the following 48 h	28/46 = 61%
Hutchins 2023 [27]	USA	2015–2021	Retrospective cohort	199	103	96	56.2 ± 14.6	53.1 ± 16.2	67	71	NR	NR	NR	Cardiogenic shock	Successful decannulation was defined as survivall without relapse to mechanical circulatory support or heart transplant within 30 days	103/199 = 51.8%
Joseph 2019 [28]	USA	NR	Retrospective cohort	30	NR	NR	NR	NR	NR	NR	NR	NR	NR	NR	NR	NR
Kellnar 2024 [29]	Germany	2021–2023	Prospective cohort	12	7	5	53.0 (IQR 47.0; 60.0)	58.0 (IQR 57.5; 67.0)	NR	NR	26.9 (IQR 25.2; 30.0)	24.9 (IQR 23.7; 26.9)	NR	Cardiogenic shock	Not requiring further mechanical circulatory support within 30 days	7/12 = 58%
Kim 2021 (A) [30]	South Korea	2016–2018	Prospective cohort	92	64	28	60 ± 12	59 ± 12	48	21	24 ± 3	25 ± 4	NR	CS post-AMI (48%) Ischemic cardiomyopathy (48%), Other (4%)	ECMO removal and not requiring further MCS over the following 30 days	64/92 = 70%
Kim 2021 (B) [31]	South Korea	2016–2019	Prospective cohort	79	50	29	63 ± 13	58 ± 12	41	23	25 ± 3	25 ± 4	NR	Post-MI CMP (52%), Idiopathic dilated CMP (18%), Fulminant myocarditis (4%), Stress-induced CMP (4%)	Successful removal of V-A-ECMO and no further mechanical circulatory support in the following 30 days	50/79 = 63%
L’Acqua 2019 [32]	Italy	2012–2018	Retrospective cohort	98	49	49	NR	NR	NR	NR	NR	NR	NR	NR	Patient weaned from V-A ECMO	49/98 = 50%
Lee 2023 [33]	South Korea	2017–2019	Retrospective cohort	55	38	17	NR	NR	NR	NR	NR	NR	NR	NR	NR	38/55 = 69%
Lim 2019 [34]	South Korea	2010–2018	Retrospective cohort	122	72	50	57.8 ± 15.0	NR	NR	NR	NR	NR	NR	NR	NR	72/122 = 59%
Matsumoto 2018 [35]	Japan	1995–2014	Retrospective cohort	37	22	15	44 ± 32	40 ± 31	13	8	21 ± 3	22 ± 4	NR	Myocarditis (100%)	ECMO removal	22/37 = 59%
Mongkolpun 2019 [36]	Belgium	NR	Retrospective cohort	22	12	10	NR	NR	NR	NR	NR	NR	NR	CS post-AMI (64%), post-cardiotomy (14%), Myocarditis (14%), PE (8%)	ECMO removal and HD Stabilization without the need to increase the Vasopressor dose within 24 h	12/22 = 55%
Naruke 2010 [37]	Japan	1996–2008	Retrospective cohort	25	18	7	54 ± 14	49 ± 18	8	5	NR	NR	3	Myocarditis (52%), CS post-AMI (36%), ACHF (12%)	ECMO weaning	18/25 = 72%
Naruke 2012 [38]	Japan	NR	Retrospective cohort	30	NR	NR	NR	NR	NR	NR	NR	NR	NR	NR	V-A-ECMO weaned of without severely deteriorated cardiac output indicated by ETCO2 < 10 mmHg or LVET < 100 ms	NR
North 2018 [41]	USA	2012–2017	Retrospective cohort	60	42	18	NR	NR	NR	NR	NR	NR	NR	NR	V-A-ECMO decannulation	42/60 = 70%
Ouazani 2019 [42]	USA	NR	Prospective cohort	12	9	3	NR	NR	NR	NR	NR	NR	NR	NR	ECMO removal without requiring any further MCS	9/12 = 75%
Punn 2019 [39]	USA	2010–2018	Prospective cohort	63	25	38	58 ± 21	32 ± 40	16	20	NR	NR	NR	Congenital heart defect (63%), Myocarditis (15%), Idiopathic dilated cardiomyopathy (14%), Sepsis (2%), Others (6%)	wean within 48 hours of assessment and survived without ventricular assist devices or orthotopic heart transplantation	25/63 = 40%
Sawada 2021 [40]	Japan	2013–2017	Retrospective cohort	50	24	26	76 ± 21	64 ± 29	20	17	23 ± 6	23 ± 6	5	CS post-AMI (54%), FM (24%), CMP (10%), other heart disease (12%)	ECMO removal and survival beyond 30 days without needs for further MCS	24/50 = 48%
Stull 2013 [41]	USA	2010–2013	Retrospective cohort	23	15	8	NR	NR	NR	NR	NR	NR	NR	ARDS (100%)	NR	15/23 = 65%
Sugiura 2019 [42]	Japan	2012–2016	Retrospective cohort	55	28	27	64 ± 14	68 ± 16	21	25	25 ± 4	25 ± 5	NR	CS post-AMI (100%)	ECMO removal	28/55 = 51%
Sugiyama 2019 [43]	Japan	2011–2018	Retrospective cohort	74	37	37	NR	NR	NR	NR	NR	NR	NR	NR	Patient survives more than 48 h after the removal of cannulas of ECMO	37/74 = 50%
V-ArodomV-Anichkul 2023 [44]	Thailand	2018–2021	Retrospective cohort	57	46	11	NR	NR	NR	NR	NR	NR	NR	Cardiogenic shock	NR	46/57 = 81%
Voigt 2022 [45]	Germany	2017–2020	Retrospective cohort	40	16	24	51 ± 17	60 ± 15	10	18	28 ± 6	27 ± 6	NR	Cardiac arrest (40%), Cardiogenic shock (60%)	V-A-ECMO decannulation and subsequent discharge	16/40 = 40%
Watanabe 2023 [46]	Japan	2010–2016	Retrospective cohort	41	17	24	70.6 ± 13.2	68.0 ± 14.5	12	17	NR	NR	NR	NR	Survival for more than 24 hours after V-A-ECMO withdrawal without requiring reintroduction	17/41 = 41%

ACHF, acute on chronic heart failure; AMI, acute myocardial infarction; ARDS, acute respiratory distress syndrome; CA, cardiac arrest; CMP, cardiomyopathy; CPR, cardiopulmonary resuscitation; CS, cardiogenic shock; ECMO, extracorporeal membranous oxygenation; FM, fulminant myocarditis; MCS, mechanical cardiac support; NR, not reported; PE, pulmonary embolism; VF, ventricular fibrillation; VT, ventricular tachycardia.

#### Biomarkers.

**Fig 3:**
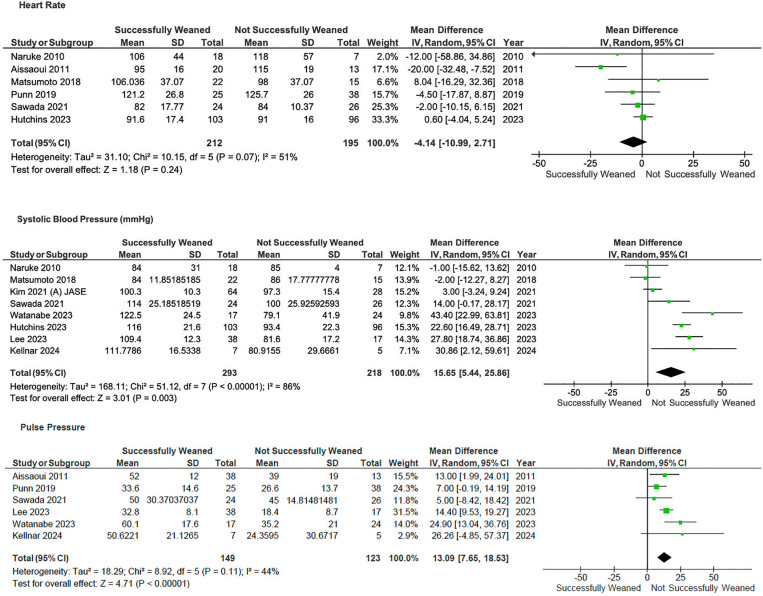
Forrest plot of comparison of haemodynamic parameters on V-A ECMO Weaning.

Markers of organ damage were inversely associated with weaning success—Fig 3. Lower levels of creatinine kinase (CK-MB) (MD −4.1, 95%CI −6.6 to −1.6, p = 0.001; I^2^ = 24%), lactate at admission (MD −3.2, 95% CI −4.8 to −1.5, p < 0.001; I^2^ = 90%), and lower levels of alanine aminotransferase (ALT) (MD −36.7, 95%CI −65.5 to 7.9, P = 0.01; I^2^ = 0%) at the time of weaning were associated with weaning success. Too few studies reported NT-ProBNP or Troponin to enable analysis.

#### Haemodynamics.

**Fig 4:**
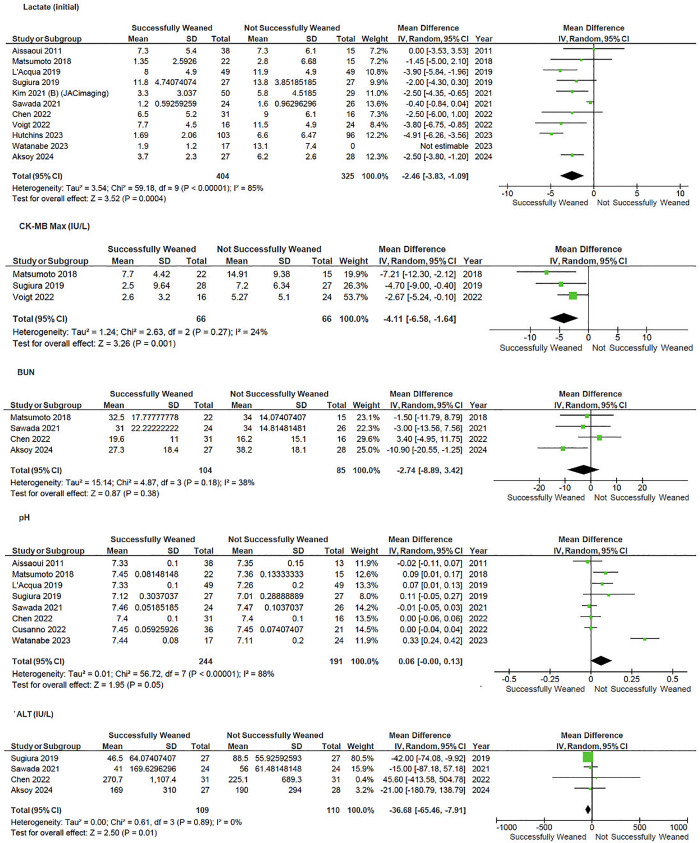
Forrest plot of comparison of laboratory parameters on V-A ECMO.

Patients with higher pulse pressure (MD 13.1, 95%CI 7.7–18.5*,* p < 0.001; I^2^ = 44%) and systolic blood pressure (MD 15.7, 95%CI 5.4–25.9, p < 0.001; I^2^ = 86%) were associated with weaning success—Fig 4.

#### Echocardiography.

**Fig 5:**
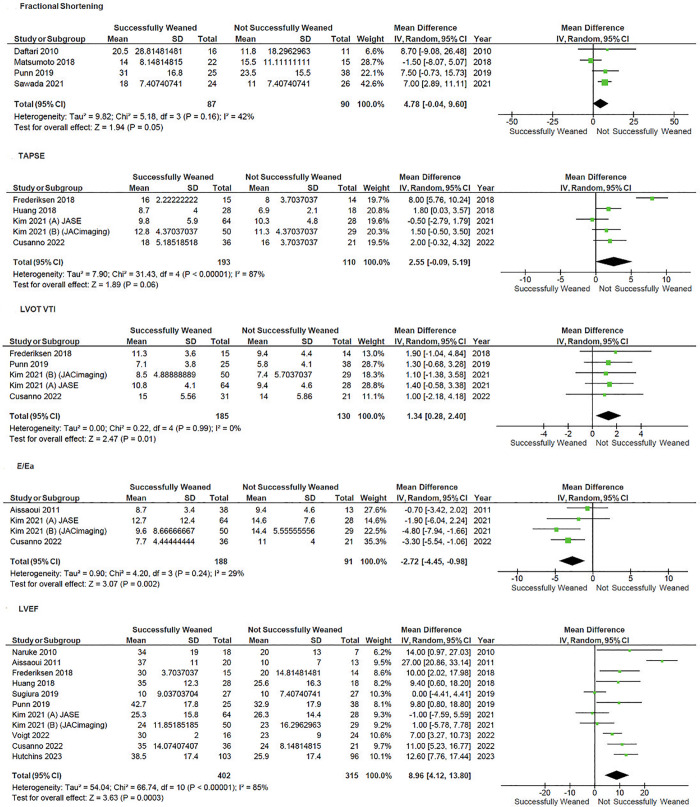
Forest plot of the comparison of different echocardiographic parameters on V-A ECMO weaning.

Patients with higher left ventricular ejection fraction (LVEF) at time of weaning (MD 9.0, 95% CI 4.1–13.8; p < 0.001; I^2^ = 85%), left ventricular outflow tract velocity time integral (LVOT VTI) (MD 1.35, 95% CI 0.28–2.40, p = 0.01; I^2^ = 0%), E/Ea (MD −2.72, 95% CI −4.45 to −0.98, p = 0.002; I^2^ = 29%) were associated with weaning success—Fig 5.

#### Multi-variable adjusted: Risk predictors.

Thirteen studies provided multi-variable adjusted analysis to identify predictors of successful weaning. Covariates tested varied widely between studies; only lack of renal failure or CRRT during ECMO, and post-weaning lactate, reported by more than one study as predictors of successful ECMO weaning—Table 2.

**Table 2:** Risk factors predictive of successful ECMO weaning from multi-variable logistic regression models.

Baseline characteristics	Study
Normal RV function	Puerto [47]
No pre-existing ischemic cardiopathy	Cusanno [9]
Post PCI TIMI flow	Sugiura [42]
**While on ECMO**	
No CRRT	Kim [31]
No need for LV venting	Kim [31]
10% improvement of tricuspid S’ during ECMO flow study	Kim [30]
Any improvement of lateral e’ during ECMO flow study	Kim [30]
ECMO duration	Punn [39]
Implantation-weaning test delay < 7 days	Cusanno [9]
**Echo findings on weaning**	
VTI	Lim [48], Punn [39]
LV EF	Punn [39]
Corrected LV ejection time/PAWP	Sawada [40]
Tricuspid annular S’/RSVP >0.33	Kim [31]
RV EF	Huang [26]
Normal RV function	Puerto [47]
RV free wall strain	Huang [26]
RV FAC	Huang [26]
**Hemodynamics**	
HR on day of decannulation	Liu [49]
MAP at weaning	Lim [48]
MAP at 4hrs	Sugiura [42]
Post-test SBP >120 mmHg	Cusanno [9]
CVP on day of decannulation	Huang [26]
**Post de-cannulation**	
Lactate at 12hrs	Chen [19]
Lactate at 24hrs	Sugiura [42]
Lower vasoactive-inotropic score 24hrs post cannulation	Dunton [22]
Improvement of RV systolic function >24hrs after decannulation	Puerto [47]

CRRT, continuous renal replacement therapy; CVP, central venous pressure; ECMO, extracorporeal membrane oxygenation; EF, ejection fraction; FAC, fractional area change; HR, heart rate; LV, left ventricle; MAP, mean arterial pressure; PAWP, pulmonary artery wedge pressure; PCI, percutaneous coronary intervention; RV, right ventricle; SBP, systolic blood pressure; TIMI, Thrombolysis in Myocardial Infarction score; VTI, velocity time integral.

Additional sensitivity analyses removing studies that included paediatric patients did not reveal any significant differences—S4 Table.

#### Study quality.

A total of 34 publications were eligible for quality assessment, of which 12 publications were of poor quality, i.e., ‘critical’ risk of bias (Fig 2). Many studies did not provide detailed protocols, limiting methodological assessment, appraisal of the confounding effect of intervention and bias in selection of participants into the study.

## Discussion

In this systematic review of predictors of V-A ECMO weaning success 34 predominantly small observational, studies were identified. On pooled analysis, lower levels of biochemical markers of end-organ perfusion or injury (lactate, CK-MB and ALT), haemodynamic (pulse pressure and systolic blood pressure) and echocardiographic indicators of myocardial function (LVEF, LVOT VTI, E/Ea) were associated with successful weaning form V-A ECMO.

To our knowledge this review is only the second to systematically assess predictors of V-A ECMO wean success. The first, in adult patients with specifically cardiogenic shock or cardiac arrest identified similar results to our review, with lower creatine kinase and lactate levels, and higher LVEF being predictors for successful weaning from V-A-ECMO [10] Other, non-systematic, reviews have reported also reported lower creatine kinase and lactate levels, and higher LVEF and LVOT VTI being predictors for successful weaning from V-A-ECMO [50]. Several other V-variables used in clinical practice [9] Troponin, NT-ProBNP, RV to PA coupling indices were not identified owing to limited numbers of studies and patients reported with these.

Despite significant heterogeneity, small sample sizes and a significant risk of bias, there are some conclusions that can be drawn from this review and the available literature. First, determination of likely weaning success, should consider multiple variables and not be focussed on one individual predictor. Factors associated with success (or failure), were present across clinical, biochemical, haemodynamic and echocardiographic parameters and clinicians should avoid relying on one variable over the complete picture of the patient. Second, initial severity of illness (e.g., lactate), markers of end-organ perfusion, and then recovery of such are important considerations in attempting to wean [42,51,52]. Further, absolute cut offs for specific variables, e.g., LVEF or LVOT VTI to predict weaning success vary between studies, are based empirical clinical weaning protocols [50,53] and therefore cannot yet be elucidated. Overall restitution and improvement of the overall clinical state of the patient as well as cardiac function is likely key to successful weaning rather than a specific variable or level of a variable.

Formal weaning or “ramp” studies that assess haemodynamic and echocardiographic changes to alterations to ECMO flows protocols are recommended [54] but as yet no standardised protocols exist, are only variably reported in ECMO trials, and are not formally assessed in this systematic review. However, they are critical tools to assess the response of cardiac function to reduction, and then removal, of mechanical circulatory support [9,55]. Future prospective clinical trials should publish weaning strategies and protocols to enable further assessment and comparison of strategies.

### Limitations

Our review is limited by the lack of large high-quality trial, with all included studies consisting of observational studies with small sample sizes and these small trials were used to investigate widely varying interventions amongst this population group, often performed without covariate adjustment. However, we completed a comprehensive review of the literature including all commonly used variables for V-A ECMO weaning. The inclusion criteria of our study were broad and included adult and paediatric patients and any aetiology leading to V-A ECMO support. It is possible that paediatric and adult patients differ in weaning factors. Additional analysis excluding studies that included paediatric patients showed no significant change in outcomes reported. Further, V-A ECMO patients by their very nature are heterogenous and a wide array of prognostic variables are utilized by clinicians in clinical practice, a wider approach to inclusion criteria increases sensitivity and generalisability of findings. Micro-circulation indices were not assessed but are not in uniform clinical practice which was our focus. The underlying aetiology leading to requirement for V-A ECMO support was not assessed and may impact timing of weaning, weaning success and need for durable ventricular support, e.g., VAD.

## Conclusions

In patients requiring V-A ECMO support, multiple biochemical, haemodynamic and echocardiographic parameters of recovery, rather than a single variable should be used to guide appropriateness for weaning. Further larger studies are required to determine optimal weaning strategies.

## Supporting information

S1 Appendix Prisma checklist. (DOCX)

S2 Appendix Search strategy. (DOCX)

S1 Table Literature search and decisions. (XLSM)

S2 Table Data extraction. (XLSX)

S3 Table Risk of bias. (XLSX)

S4 Table Paediatric sensitivity analysis. (DOCX)
