# Supplementary material for: Predictors of successful weaning from veno-arterial extracorporeal membrane oxygenation (V-A ECMO): A systematic review and meta-analysis
Source: PLoS One. 2025 Mar 19;20(3):e0310289. doi: 10.1371/journal.pone.0310289 (PMC11922212; doi:10.1371/journal.pone.0310289)
Supplement: S4 Table — (DOCX) [file pone.0310289.s006.docx]

|  | All patients | Adult patients only |
| --- | --- | --- |
| Creatinine kinase (CK-MB) | MD -4.1, 95%CI -6.6 – -1.6, p=0.001; I^2^ = 24% | No change |
| Lactate on admission | MD -3.2, 95% CI -4.8 - -1.5, p<0.001; I^2^ = 90% | MD -3.3, 95% CI -5.4 – -1.3, p = 0.001; I^2^ = 92% |
| Alanine aminotransferase | MD -36.7, 95%CI -65.5 – 7.9, P=0.01; I^2^ = 0% | MD -37.6, 95%CI -66.7 – 8.6, P=0.01; I^2^ = 0% |
| Pulse pressure | MD 13.1, 95%CI 7.7 – 18.5, p<0.001; I^2^ = 44% | MD 15.0, 95%CI 9.4 – 20.6, p<0.001; I^2^ = 27% |
| Systolic blood pressure | MD 15.7, 95%CI 5.4 – 25.9, p<0.001; I^2^ = 86% | No change |
| Left ventricular ejection fraction | MD 9.0, 95% CI 4.1 – 13.8; p<0.001; I^2^ = 85% | MD 8.9, 95% CI 3.7 – 14.1; p<0.001; I^2^ = 87% |
| Left ventricular outflow tract velocity time integral | MD 1.35, 95% CI 0.28 – 2.40, p=0.01; I^2^ = 0% | MD 1.35, 95% CI 0.10 – 2.61, p=0.04; I^2^ = 0% |
| E/Ea ratio | MD -2.72, 95% CI -4.45 – -0.98, p=0.002; I^2^ = 29% | No change |

Supplementary Table XXX. Meta-analysis results with all patients, and exclusion of three paediatric studies (Aksoy 2024, Chen 2022, Punn 2019). CI, confidence interval, MD, mean difference.
